# A Pathologist's Survey of Superficial Tumors Removed in a Casualty Department
*Originally written for the Report of the South Western Regional Cancer Records Bureau and reproduced with permission of the Director.This material came from the Casualty Department of the Bristol Royal Infirmary.


**Published:** 1960-01

**Authors:** T. F. Hewer

**Affiliations:** Professor of Pathology, University of Bristol.


### A PATHOLOGIST'S SURVEY OF SUPERFICIAL TUMORS REMOVED IN A CASUALTY DEPARTMENT*

T. F. HEWER

*Professor of Pathology, University of Bristol.*

The Casualty Department of a large hospital provides a fascinating collection of clinical material, ranging as it does from street accidents and sudden severe medical or surgical illness to the patient who wishes to have a minor cutaneous blemish removed for cosmetic reasons. It is especially the people in the last category and those with less trivial but apparently minor superficial lesions who provide a wealth of varied problems for the diagnostic histology department. Fortunately it is the custom to send all such tissue for examination.

From the pathologist's point of view these superficial lesions provide a most interesting diagnostic exercise. Some idea of the variety can be obtained by scrutiny of the list (Table 1) which represents the specimens received in twelve months. They are listed not in order of frequency but in groups of more or less related conditions; the number of examples of each is indicated alongside.

TABLE I

| Diagnosis | No. of Specimens |
|---|---|
| Inflammatory | 23 |
| Epithelial hyperplasia, not primarily inflammatory | 5 |
| Epidermal cyst | 23 |
| Keratosis senilis | 2 |
| Squamous papilloma | 38 |
| Cornu cutaneum (sebaceous wart) | 2 |
| Squamous carcinoma | 14 |
| Basal cell carcinoma | 22 |
| Verruca vulgaris | 8 |
| Verruca plantaris | 2 |
| Molluscum sebaceum ( = pseudocarcinomatosum) | 4 |
| Benign calcified "epithelioma" | 4 |
| Molluscum contagiosum | 1 |
| Fibro-epithelial polyp | 9 |
| Benign sweat gland tumour | 1 |
| Malignant sweat gland tumour | 1 |
| Secondary carcinoma | 5 |
| Benign melanoma | 24 |
| Malignant melanoma | 6 |
| Blue naevus | 1 |
| Benign juvenile melanoma | 1 |
| Histiocytoma (sclerosing angioma) | 4 |
| Haemangioma | 18 |
| Lymphangioma | 1 |
| Lipoma | 22 |
| Neurofibroma | 15 |
| Sarcoma (five being neurofibrosarcoma) | 6 |
| Leiomyoma | 2 |
| Keloid | 5 |
| Foreign body granuloma | 2 |
| Other Lesions | 11 |
| Total | 282 |

* Originally written for the Report of the South Western Regional Cancer Records Bureau and reproduced with permission of the Director.

This material came from the Casualty Department of the Bristol Royal Infirmary.

The epidermal cysts included eight sebaceous cysts but the remainder were a very mixed bag; there were a few implantation dermoids but several were simple cysts with no sebaceous glands alongside, lined by degenerate squamous epithelium and containing desquamated keratin and amorphous debris which did not appear sebaceous in the gross. Some of these were doubtless due to obstruction of sweat glands, irregular hair follicles and other disorders of epithelial adnexa and all were unmistakably benign.

An observation often made in the laboratory is that a large number of excised cutaneous lesions are described on the report form as sebaceous cysts. They are generally sent unopened—and we prefer to receive them like that—and in consequence it is not easy to determine at the time of excision whether they are cystic or solid. This point is worth mentioning because there must be occasions in practice where a rounded subcutaneous tumour which appears cystic may in fact be a carcinoma metastasis or one of the many forms of sub-epidermal basal cell carcinoma.

The squamous papillomata seldom gave much difficulty in diagnosis and the same was true of the benign calcified "epitheliomata" although the latter condition is notoriously apt to be misleading and trap the unwary into making a false diagnosis of malignancy.

Molluscum sebaceum or, as it is often called, kerato-acanthoma or—another synonym—molluscum pseudocarcinomatosum is a condition that always causes the histologist anxiety. It grows rapidly, in a few weeks, and forms a rounded projecting epithelial tumour with central keratinization. Quite miraculously it regresses in a few more weeks and leaves a scar. Histologically it is often impossible to be certain that it is not a true squamous carcinoma and consultation with the clinician is essential for an accurate diagnosis.

The melanomata are an important and interesting group. Some of the benign ones were removed for cosmetic reasons but most on suspicion of malignancy. The benign juvenile melanoma, of which only one example appeared in the year under review, is sometimes mistakenly reported as malignant on account of its quite alarming histological appearance.

Among the more difficult specimens for diagnosis were the small lesions removed from eyelids. The ophthalmic surgeon is faced with peculiar difficulty in removing the whole of a lesion on the lid margin and when the excision is for a diagnostic biopsy the tendency is, very properly, to be conservative. Not infrequently the specimen in such cases consists of a slice of keratinizing epidermis with no indication of the junction with the dermis.

Routine histological examination of every piece of tissue removed in Casualty, in Out-Patients or in the general practitioner's surgery is a most necessary precaution. One hopes that the Regional Cancer Records Bureau will always be informed when one of these lesions proves to be malignant or equivocal because a follow-up in such cases is of the highest importance. This is perhaps particularly true in the case of the basal cell carcinomata, since they may not have been completely removed, and the malignant melanomata because these latter are not necessarily as malignant as their reputation suggests and careful observation with prompt dissection of lymph nodes at the first appearance of a recurrence may well be a life-saving measure.

I think the most important point arising from a survey of material such as this is that there are numerous conditions on the borderline of malignancy: many of them are reported as benign and even when uncertainty is expressed by the pathologist I fear that the case is not reported to the Cancer Bureau. I am sure a lot of patients are thus lost sight of and I suggest that an improvement in the procedure of follow-up is overdue.

